# Socioeconomic differences in labour market attachment after breast cancer: a population-based matched cohort study

**DOI:** 10.1007/s00520-026-10411-9

**Published:** 2026-02-17

**Authors:** Cathrine F. Hjorth, István Bakos, Deirdre Cronin-Fenton

**Affiliations:** Department of Clinical Epidemiology, Department of Clinical Medicine, Aarhus University and Aarhus University Hospital, Aarhus N, Denmark

**Keywords:** Social benefits, Cohort study, Socioeconomic position, Survivorship, Sick leave, Disability, Breast neoplasms

## Abstract

**Purpose:**

Breast cancer survivors face challenges maintaining labor market attachment, potentially impacting their health and economy. Yet, the broader public health impact on how labor market attachment patterns compare with women in the general population by socioeconomic group and disease stage remains unknown. Most previous studies have only included employed women, limiting their relevance for broader population-level planning. This study aimed to address these gaps.

**Methods:**

Using Danish registries, we identified women aged 25–55 years diagnosed with stage I–III breast cancer during 2002–2018. For each, we matched 10 comparison women on age, region, and surgery date (index). We assessed sick leave, workforce detachment, and disability pension up to 10 years, emigration, death, or 31 December 2023. We calculated incidence rates and used negative binomial regression to estimate rate differences and ratios, stratified by stage, education, cohabitation, and a social vulnerability index (rSVI, range 0–14, where rSVI < 5 indicated lowest vulnerability).

**Results:**

Among 13,443 breast cancer survivors and 134,430 comparisons, survivors more often had long education, a partner, and a lower rSVI. Breast cancer survivors had higher incidence rates of sick leave 0–2 years after index, but slightly lower rates of workforce detachment and disability pension. From year 3 onwards, rates of all outcomes were slightly higher in breast cancer survivors. Rates of workforce detachment and disability pensions were particularly elevated among survivors with advancing stage and lower socioeconomic position. Notably, the relative estimates were greatest among women with a partner, medium/long education, or lower rSVI.

**Conclusion:**

By assessing labour market outcomes among all breast cancer survivors regardless of their employment status, this study provides population-relevant evidence on the long-term public health consequences of breast cancer. While women with advanced stage and lower socioeconomic position had the highest incidence of work force detachment and disability pension, the greatest relative impact of breast cancer occurred among high-resource survivors—likely reflecting their lower baseline risk.

**Supplementary Information:**

The online version contains supplementary material available at 10.1007/s00520-026-10411-9.

## Introduction

Breast cancer is the most common cancer worldwide, with approximately 2.3 million new cases diagnosed each year [1]. Advancements in diagnosis and treatment have led to increased survival rates. However, survivors may face challenges maintaining labor market affiliation due to the psychological and physical sequelae of the disease and its treatments [2, 3]. This may have long-lasting financial consequences at a personal and societal level [4–9]. As one-third of breast cancer cases occur in working-age women [10], understanding how survivors vary in terms of social benefits is essential to public health planning. A critical point remains to identify potential socioeconomic differences, which is essential to guide follow-up strategies, reduce reliance on social benefits, and facilitate return-to-work initiatives. In addition, adverse effects of systemic breast cancer treatments—such as chemotherapy and endocrine therapy—as well as cancer stage at diagnosis, may compromise a woman’s ability to sustain workforce attachment.

For at least 10 years after diagnosis, breast cancer survivors have a higher risk of disability pension, unemployment, and sick leave than women in the general population [7, 11]. Social differences also appear important, as survivors who lived alone or had shorter education have a higher frequency of disability pensions [8]. These differences may partly reflect the consequences of the cancer and its treatment [11]. However, they may also arise from background socioeconomic and health-related factors that influence labour-market patterns in the general population. Yet, the extent to which these social gradients among breast cancer survivors exceed the baseline social gradients observed in the general population remains unclear. Previous studies examining unemployment, disability pension and/or sick leave in breast cancer survivors were in historical cohorts [4, 12–14], had a short follow-up (2–5 years) [4, 11, 12, 14, 15], or focused on employed women who generally have better socioeconomic resources [4, 5, 12, 14, 16]. Such restrictions likely underestimate the incidence of disability pension, which often occurs after prolonged sick leave or workforce detachment. Moreover, studies included women with metastatic disease who are likely to have considerably higher uptake of social benefits [4, 5, 7, 17].

Using Danish population-based registries, we evaluated socioeconomic differences in sick leave, workforce detachment, and disability pensions among breast cancer survivors compared with women from the general population. We also assessed the influence of stage.

## Material and methods

This study was approved by the Danish Data Protection Agency (record serial number: 2022–0367531, 2979) and the Danish Healthcare Quality Institute (DHQI). It adheres to the General Data Protection Regulations.

### Setting

The Danish welfare system provides citizens with free education, social support, tax-funded retirement, and universal access to healthcare [18]. If an individual becomes temporarily or permanently unable to work, the state offers financial assistance to their employer or directly to the individual, often conditional on active participation in labor market reintegration programs. In case of permanent loss of work capacity, individuals can apply for a federal disability pension. The monthly amount depends on the calendar year the pension was granted and the woman’s cohabitation status and other income sources. If the woman herself or her spouse has an income beyond a certain level, the disability pension will be reduced. If the work capacity is either partly reduced or judged to be temporary, the person can apply for “flex job”—a government-supported position with reduced working hours and responsibilities tailored to the individual’s work capacity. In 2013, a structural modification of the Danish Disability Pension Act altered the eligibility criteria. Individuals under the age of 40 became largely ineligible for disability pension, and the overall qualification criteria were tightened [13].

The Danish Breast Cancer Group (DBCG) provides diagnostic and treatment guidelines for breast cancer in Denmark [19]. The DBCG clinical database registers all women diagnosed with a first primary non-distant metastatic breast cancer, including patient, tumor, and treatment characteristics, and clinical follow-up.

### Study population

The breast cancer cohort encompassed premenopausal women aged 25–55 years, diagnosed with non-distant metastatic breast cancer during 2002–2018, identified from the DBCG database, regardless of labor market affiliation at diagnosis.

The comparison cohort included women from the general population with no prior breast cancer surgery matched on index date (date of primary surgery in the breast cancer cohort), age, and region of residence. Up to 10 comparisons were matched to each woman in the breast cancer cohort. Woman who later developed breast cancer contributed person-time to the breast cancer cohort.

All included women had lived in Denmark ≥ 1 year before the index date. Women who were students in the month before diagnosis/index were excluded, as these women have not yet entered the labor market and disruption in their education path would not be detected (Appendix 1: Figure A.5).

### Data sources and covariates

Registries were linked on an individual level via a unique personal identifier assigned to all Danish residents at birth or upon immigration [18]. For the breast cancer cohort, we collected clinical information from the DBCG database, including intention-to-treat (ITT) chemotherapy and endocrine therapy, surgery type, estrogen receptor (ER) status, human epidermal growth factor receptor 2 status, disease stage and breast cancer recurrence (Appendix 1: Table A.1). Based on records in the Danish National Patient Register and redeemed prescriptions in the Danish Prescription Register, we summarized psychiatric and somatic comorbidities using the Nordic Multimorbidity Index (Appendix 1: Table A.2) [20].

Following the SEPLINE recommendations [21], we classified women as either living alone or cohabiting using data from the Danish Civil Registration System, and women as employed or not employed using the Integrated Database for Labour Market Research. We retrieved information on educational attainment from the Danish Population’s Education Registry categorized as short (primary education), medium (upper secondary education), and long education (tertiary education). In addition, we scored each woman according to a registry-based social vulnerability index (rSVI) weighting information on marital status, ethnicity, education, household income, employment status, psychiatric and somatic comorbidity prior to the index date (Appendix 1: Tables A.3–6). The rSVI score ranges from 0–14 [22]. We categorized the rSVI into lower (0–4) and higher (rSVI ≥ 5), with the higher scores indicating less socioeconomic resources.

### Outcomes

From the Danish Register for Evaluation of Marginalisation (DREAM) [23], we collected weekly records of government-provided financial support from the index week up to 10 years after the index date. We examined three outcomes (1) sick leave, (2) workforce detachment, which included social assistance, unemployment, and flex jobs, and (3) disability pensions (Appendix 1: Table A.4).

### Statistical analyses

We calculated the median and associated interquartile range (IQR) for continuous covariates and frequencies for categorical and ordinal covariates.

In both cohorts, we calculated the mean number of weeks per year spent in each social benefit category 0–2 years, 3–5 years, and 6–10 years after breast cancer surgery/index date, censoring on emigration, 10 years, retirement, breast cancer surgery in the matched comparison cohort, death, or 31 st December 2023. We used negative binomial regression models with a robust variance estimator to calculate incidence rate differences in weeks per person-year and incidence rate ratios within strata of stage, education, cohabitation, and rSVI score. The models were adjusted for age, region of residence, and the Nordic Multimorbidity Index. We repeated the analyses stratified on socioeconomic position restricting to patients who were assigned to ITT adjuvant chemo- or endocrine therapy and to women who were employed at diagnosis.

To explore the impact of the Danish Disability Pension Act reform, we plotted annual incidence rates of disability pensions per 1000 person-years in both cohorts and stratified by age (cut-off 40 years), education, cohabitation, and rSVI.

The analyses were performed in SAS version 9.4 (SAS Institute Inc, Cary, NC, USA). The forest plots were generated using RStudio version 2022.12.0 + 353.

## Results

Among the 13,443 breast cancer survivors and 134,430 comparisons, breast cancer survivors were more likely to live with a partner (77% vs. 74%), have long education (41% vs. 36%), be employed (86% vs. 82%), and were less likely to have a higher rSVI score (14% vs. 18%). In the breast cancer cohort, 20% had ER– tumors, 15% had stage III disease, 35% underwent mastectomy, 73% were assigned ITT adjuvant endocrine therapy, 91% ITT adjuvant chemotherapy, and 84% received chemotherapy (Table 1).

**Table 1:** Baseline characteristics of women diagnosed with stage I–III breast cancer during 2002–2018 and their matched comparison cohort

	Breast cancer cohort		Comparison cohort	
	*N*	%	*N*	%
Total	13,443	100.0	134,430	100.0
Age				
25–34	829	6.2	8,329	6.2
35–44	4617	34.3	46,251	34.4
45–55	7997	59.5	79,850	59.4
Calendar period				
2002–2006	3886	28.9	38,860	28.9
2007–2011	4267	31.7	42,670	31.7
2012–2018	5290	39.4	52,900	39.4
Nordic Multimorbidity Index				
< 0	299	2.2	3,906	2.9
0	7864	58.5	86,286	64.2
1–2	3063	22.8	21,396	15.9
3 +	2217	16.5	22,842	17.0
Cohabitation status*				
Cohabiting	10,310	76.7	99,435	74.0
Living alone	3130	23.3	34,955	26.0
Missing	5	0.0	40	0.0
Educational level				
Long	5440	40.5	48,679	36.2
Intermediate	5238	39.0	53,136	39.5
Short	2568	19.1	29,676	22.1
Missing	197	1.5	2,939	2.2
Social vulnerability index score				
Lower (0–4)	11,338	84.3	107,615	80.1
Higher (5 +)	1893	14.1	23,716	17.6
Missing	212	1.6	3,099	2.3
Employment status				
Employed	11,547	85.9	109,913	81.8
Not employed	1896	14.1	24,517	18.2
ER status				
ER-	2671	19.9		
ER +	10,724	79.8		
Missing	48	0.4		
HER2 status				
Negative	9060	67.4		
Positive	2311	17.2		
Not tested	136	1.0		
Missing	1936	14.4		
Stage				
Stage I	4387	32.6		
Stage II	5722	42.6		
Stage III	2005	14.9		
Missing	1329	9.9		
Histological grade				
Grade 1	2231	16.6		
Grade 2	4924	36.6		
Grade 3	3856	28.7		
Not graded	1120	8.3		
Missing	1312	9.8		
Surgery type				
Mastectomy	4732	35.2		
Breast conserving surgery	7488	55.7		
Missing	1223	9.1		
ITT chemotherapy				
No	1168	8.7		
Yes	12,275	91.3		
ITT endocrine therapy				
No	3692	27.5		
Yes	9751	72.5		

**Fig. 1:**
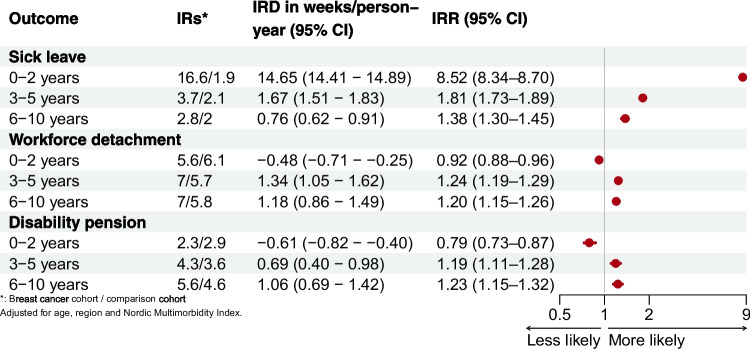
Incidence of sick leave, workforce detachment, and disability pension by follow-up period in breast cancer survivors compared with the comparison cohort

**Fig. 2:**
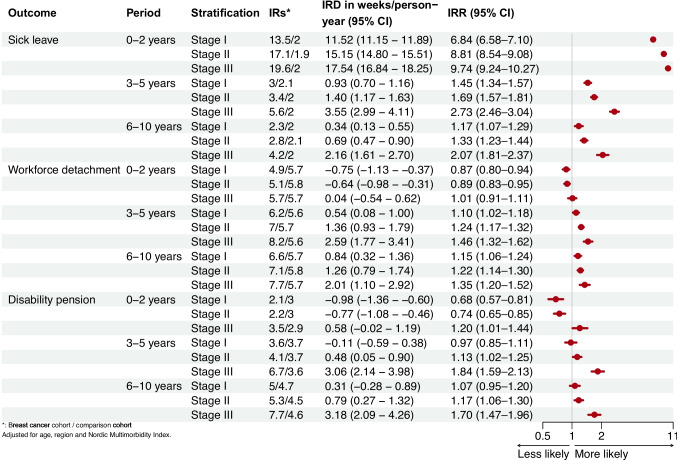
Incidence of sick leave, workforce detachment and disability pension by follow-up period in breast cancer survivors compared with the comparison cohort according to stage

The excess number of weeks on sick leave in breast cancer survivors was 14.65 (95% CI 14.41–14.89) per person-years during the first 0–2 years. Workforce detachment and disability pension rates were lower in the same period. In later periods, all outcomes were more frequent in breast cancer survivors than comparisons (Fig. 1). The number of weeks on sick leave and disability pension increased with advancing cancer stage at diagnosis throughout follow-up. In contrast, there were minimal differences in workforce detachment by stage (Fig. 2).

**Fig. 3:**
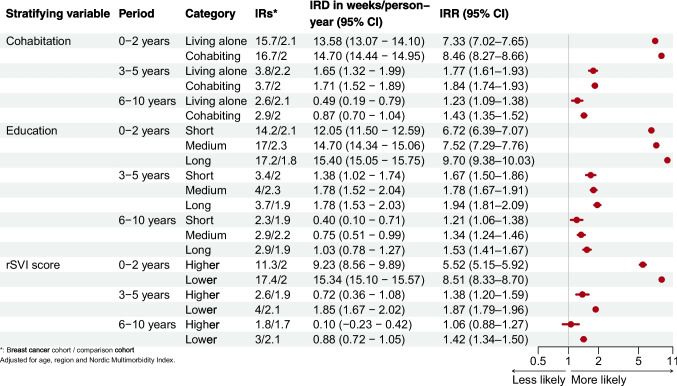
Incidence of sick leave by follow-up period in breast cancer survivors compared with the comparison cohort according to cohabitation, education, and rSVI

Across all socioeconomic strata and time periods, breast cancer survivors had increased absolute and relative rates of sick leave. During the first 2 years, breast cancer survivors’ incidence rates of sick leave were similar by cohabitation status, but higher for those with medium or long education or lower rSVI. We observed a disproportionate gradient, with lowest rate ratios among those living alone, with short education, or higher rSVI, and progressively higher rate ratios with increasing socioeconomic position (i.e., cohabiting, long education, or lower rSVI) (Fig. 3).

**Fig. 4:**
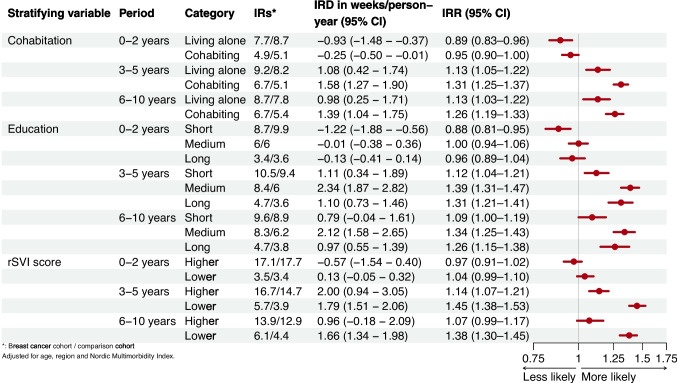
Incidence of workforce detachment by follow-up period in breast cancer survivors compared with the comparison cohort according to cohabitation, education, and rSVI

Across all socioeconomic strata, lower or similar rates of workforce detachment persisted in the initial period, likely due to the observed increase in sick leave. In the later periods, the rate differences and ratios were highest among women with high socioeconomic positions, especially those with a medium education (Fig. 4).

**Fig. 5:**
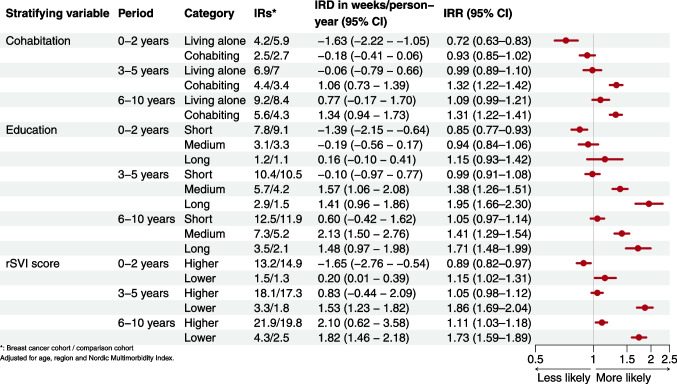
Incidence of disability pension by follow-up period in breast cancer survivors compared with the comparison cohort according to cohabitation, education, and rSVI

The lower relative ratio of disability pension 0–2 years after index differed across strata—women in the breast cancer cohort with long education and lower rSVI had slightly higher ratios than their comparisons (Fig. 5). From the third year onwards, there were minor long-term differences among those living alone, with short education or higher rSVI. In contrast, the gradients increased, with higher rates among women cohabiting, with medium or long education, and with lower rSVI. Women with medium or high education differed most from their comparisons, with 2.13 (95% CI 1.50–2.76) excess weeks of disability pension seen in those with medium education 6 to 10 years after diagnosis.

When restricting to ITT populations assigned to adjuvant chemotherapy or endocrine therapy, findings were similar (Appendix 1: Figs. A.6–2). When restricting to employed women, the results were similar, despite the sick leave and disability pension rates in those with higher rSVI which increased and decreased respectively. Importantly, employment status is one of the components of the rSVI. Thus, individuals who ranked high in the original cohort because they were unemployed were excluded by design in these analyses. As a result, the higher rSVI scores within the employed-only sample instead reflect other social challenges than unemployment (Appendix 1: Fig. A.3–8).

### Changes in disability pension uptake

The immediate effect of the 2013 reform was a decrease in the disability pension uptake in both cohorts, although the downward trend appeared to begin before the reform (Appendix 1: Fig. A.9). From 2016, the incidence gradually increased until 2020, before dropping again in 2021. In the breast cancer cohort, those aged < 40 years had slightly higher incidence of disability pensions before the reform than those aged ≥ 40 years. This was opposite in the comparison cohort. After the reform, the incidences were higher among the oldest women in both cohorts. The reform nearly eliminated the pre-reform social differences observed by cohabitation status, educational level, and rSVI. However, 7 years after the reform, the gaps were close to those observed before the reform (Appendix 1: Fig. A.10).

## Discussion

In both cohorts, women living alone, with short education, or with higher rSVI scores had the highest incidence rates of workforce detachment and disability pensions, whereas an opposite gradient was seen for sick leave. The relative rates were more pronounced among women with higher socioeconomic position. Those women with more advanced stage at breast cancer diagnosis had higher rates of sick leave and disability pensions.

Our findings suggest a socioeconomic paradox, where cohabiting women and women with higher socioeconomic position experience greater relative consequences of breast cancer, i.e., breast cancer survivors with higher socioeconomic position differed more from their comparisons, whereas differences were smaller for women with lower socioeconomic position. This was also seen when we restricted our analyses to employed women and so cannot be explained by sick leave eligibility. The opposite gradient –especially seen for disability pension uptake– in lower socioeconomic groups could reflect a ‘ceiling effect’, where baseline rates are already elevated and any additional impact of breast cancer is therefore marginal. Our findings support this interpretation, suggesting that women with lower socioeconomic position face greater challenges in labor market participation, health, or both. For those with medium or long education, such changes in their labor market attachment may have greater relative financial impact than seen in those with short education. Supporting this, a Danish study of long-term financial consequences of breast cancer found that income losses persisted for a longer period among women with long education [24].

Another contributing factor could be that women with medium or long education may be more likely to work in jobs that are cognitively demanding or involve higher responsibility. These roles can be more difficult to maintain or return to after breast cancer [25, 26]. On the other hand, women with short education may be more likely to obtain physically demanding jobs, which also is a known barrier for returning to work [27].

A previous study of breast cancer patients diagnosed during 2000–2004 in Denmark also showed a greater short-term absolute impact of breast cancer on labour market participation among women with short education [17], but increasing relative impact in those with longer education during the third year after cancer diagnosis. However, these findings were restricted to women who survived 3 years and were prone to selection and immortal time bias [28]. Our observed increasing risk of sick leave and disability pension with advancing disease stage aligns with previous studies examining this association up to 5 years after diagnosis [4, 12, 15]. Yet, our study extends these findings in a contemporary cohort, and with longer follow-up, highlighting the short and long-term consequences of breast cancer. Taken together, these findings may reflect greater symptom burden and lasting psychological sequelae in women with more advanced cancer, even many years after diagnosis [29]. Moreover, as sick leave can often lead to subsequent disability pension [30], the higher levels of sick leave observed 6–10 years after index date indicate that the risk of disability pension persists beyond the follow-up available in the current study.

Our restriction to the ITT populations yielded consistent results. Previous studies have shown that adjuvant therapies substantially increase the risk of sick leave up to 5 years following breast cancer diagnosis, compared with women who did not receive such therapies [11, 14]. Such an effect likely extends beyond this period. Due to limited sample size, we were unable to directly compare these associations with women who were not assigned adjuvant therapies. Nonetheless, in contemporary clinical practice, patients comparable to our cohort are recommended these therapies, with endocrine therapy reserved for those with ER + tumors.

While the Danish Disability Pension Act reform temporarily resulted in a reduced uptake of the disability pension, the pre-reform socioeconomic differences re-emerged over time. This may indicate that the reform kept higher socioeconomic groups in the workforce but had less impact in underserved populations. This aligns with previous research conducted among cancer patients and matched controls, which documented lower relative differences in disability pensions post-reform up to 5 years after a cancer diagnosis, particularly in high socioeconomic groups [13].

### Methodological considerations

The strengths of our study include comprehensive data on demographics, socioeconomic position, cancer characteristics, and social benefit receipt, all of which have high validity [23, 31–33]. The information on social benefits is highly detailed, enabling us to distinguish sick leave, disability pensions, and other workforce detachments from one another. Moreover, our matched design aimed to reduce confounding from regional differences and age, while our stratified analyses allowed us to examine differences across socioeconomic groups.

Several issues should be considered when interpreting our findings. The rSVI is a weighted index. While such indices can be useful for identifying patterns at the population level, assigning a single score to represent the degree of vulnerability may be problematic. Such an aggregated measure risks obscuring individual vulnerabilities, which may not align with the composite indicators or their assigned weights. We refrained from labeling the women as vulnerable, and instead, referred to the score category. Still, one should be aware that an index can mask important drivers among the indicators included in the index. Also, it is important to note that socioeconomic indicators do not directly influence cancer outcomes, and mediating factors such as lifestyle, quality of treatment, and health-seeking behavior are also likely to play a role [34]. Unfortunately, we did not have information on these factors. Another limitation is that time-varying eligibility for some social benefits may preclude the receipt of other benefits and influence observed trends. For example, if women were permanently detached from the workforce, they would not be eligible for sick leave. This might explain why those with short education had the lowest risk of sick leave in the later periods, as these have the highest rates of workforce detachment in the initial periods. Last, we did not have access to women diagnosed later than 2018. While there have been some refinements in treatment strategies—such as a broader adoption of neoadjuvant chemotherapy for selected patients [35]—standard systemic and surgical management for the majority of patients in this population has remained consistent. Therefore, we believe our findings remain relevant and informative for current cancer care.

### Generalizability

Our findings provide valuable insights relevant to other high-income countries. With the exception of the USA, all countries included in the Organisation for Economic Co-operation and Development (OECD) provide health insurance coverage to all residents [9]. These policies are designed to mitigate financial hardship related to disease and to help reduce socioeconomic differences and the financial burden of diseases like cancer. In contrast, countries lacking these protections, such as the USA, may face more pronounced socioeconomic differences due to loss of income and health insurance following cancer [9, 36]. Strategies such as expanded health coverage and initiatives that encourage employers to support continued employment of cancer survivors have been suggested by both the National Cancer Institute and the American Cancer Society to mitigate these effects [9, 37, 38]. Our findings may be broadly applicable to survivors of other cancers that affect individuals of working age, such as hematological cancers.

### Perspectives

Some women experienced long periods of the outcomes, especially sick leave and disability, while most had no weeks with the event. Notably, the estimates from the negative binomial regression cannot be interpreted as individual-level risks or as the expected experience of a typical breast cancer survivor. Instead, these estimates quantify the average excess burden at the population level, which is highly relevant for public health planning [39]. Even modest differences in mean rates translate into substantial cumulative burden when applied to an entire survivor population. With an annual number of incident breast cancers of 5000, even 1–2 excess weeks per person-year amounts to a high excess public health impact.

By providing insight into the societal burden of breast cancer and the varying consequences among different sub-groups, our findings suggest that a “one-size-fits-all” approach to return-to-work support is unlikely to be effective. Evidence indicates that survivors with lower socioeconomic position, who may be more likely to hold physically demanding jobs with limited baseline flexibility, may benefit from flexible work schedules, while highly educated survivors—who often have cognitively demanding roles—may require tailored support such as workload adjustments, modified responsibilities, or psychological assistance [25, 26]. Although these examples point to potential strategies, the evidence on effective interventions remains limited [40–42]. This calls for further research to identify strategies to improve long-term work retention and well-being. Taken together, our results emphasize that interventions should be tailored to the distinct challenges faced by different socioeconomic groups to better support labor market participation among breast cancer survivors.

## Conclusion

This nationwide matched cohort study showed that breast cancer survivors had higher rates of sick leave, workforce detachment, and disability pensions than their general population counterparts, with the highest incidence rates in women with a low socioeconomic position. Paradoxically, the relative impact of breast cancer was greatest among women with a higher socioeconomic position. We also observed that more advanced disease was consistently linked to poorer labor market outcomes. These patterns persisted across the 10-year follow-up, underlining the enduring consequences of breast cancer for labor market participation. Together, our findings highlight the need for differentiated work-related support strategies for cancer survivors. Such strategies should address both the persistent structural disadvantages in lower socioeconomic groups and the relative impact of cancer among more advantaged women, while also considering the additional burden carried by those diagnosed at higher stages.

## Supplementary Information

Below is the link to the electronic supplementary material.

Supplementary file1 (DOCX 1.28 MB)

## Data Availability

The data used for this study were obtained from Danish registries and stored at secured servers at Statistics Denmark. Owing to data protection rules, the data cannot be shared. Access to the data can be obtained through approvals from Statistics Denmark, the Danish Health Data Authority, and The Danish Healthcare Quality Institute (DHQI).
