# Supplementary material for: Socioeconomic differences in labour market attachment after breast cancer: a population-based matched cohort study
Source: Support Care Cancer. 2026 Feb 17;34(3):212. doi: 10.1007/s00520-026-10411-9 (PMC12913332; doi:10.1007/s00520-026-10411-9)
Supplement: Supplementary file 1 — Supplementary file1 (DOCX 1.28 MB) [file 520_2026_10411_MOESM1_ESM.docx]

**Appendix A**

Socioeconomic differences in labour market attachment after breast cancer: A population-based matched cohort study

Cathrine F. Hjorth, István Bakos, Deirdre Cronin Fenton

## Figure A.1. Flowchart for cohort selection

All patients registered in the DBCG database with stage I-III breast cancer

N=14,029

Final breast cancer cohort

N=13,443

Matching not possible

N=27

Received student grant within 4 weeks before breast cancer diagnosis

N=226

Not resident in DK one year before breast cancer

N=98

Not aged 25–55 years

N=228

Not registered in the Population registry

N=7

## Table A.1. Covariate information

| **Covariate** | **Data source** | **Variables** | **Details on data management** | **Time of measurement** |
| --- | --- | --- | --- | --- |
| Immigration status | Danish Population Registry | Ie_type and opr_land | Immigrant if ie_type=2 and 5101<= opr_land <=5902 or ie_type =0, 1 or 3. | At index |
| Region |  | reg | Used for matching |  |
| Cohabitation |  | FAMILIE_TYPE | Cohabiting if fulfilling one of following criteria 1) being married, 2) in a registered partnership, 3) share address with person with common child, 4) living with one person of the opposite sex and age difference is <15 years, with no children, and share address. | The year prior to index year |
| Employment | The Integrated Database for Labour Market Research | Pstill/PSOC_STATUS_KODE | 11-37 / 110-138 | The year prior to index year |
| Education | The Population’s Education Register | HF_AUDD | We used the format AUDD2014_L1L2_KT to categorize into:  Short: ISCED 0–2 ~ <10 years  Medium: ISCED 3–4 ~ 10 to 14 years  Long: ISCED 5–8 ~ ≥15 years  No completed education or missing education was included as a separate group in the adjustment sets. | At index |
| Estrogen receptor status | Danish Breast Cancer Group’s clinical database | P72 | Until 1.7.2010, <10% estrogen receptor is categorized as negative. After this date, the cut-off was 1%. |  |
| Stage |  | T  n | T refers to tumor size in mm, and N to number of positive lymph nodes. These were summarized according to the TNM (tumor, node, metastasis) classification system. |  |
| Grade |  | P50, p51, p52 and p63a |  |  |
| Surgery type |  | M45 | If M45= 1 or 3 then Mastectomy, if M45=2 or 4 then lumpectomy. |  |
| HER2 status |  | P137 |  |  |
| ITT Chemotherapy |  | ITT_KEMO  iKE | ITT adjuvant chemotherapy, yes or no. |  |
| ITT endocrine therapy |  | ITT_ENDO  iET | ITT adjuvant endocrine therapy, yes or no |  |
| Date of breast cancer diagnosis |  | M24, M23 and M25 |  |  |
| Recurrence date |  | Ob3 ob2 ob4 |  |  |

## Table A.2. Nordic Multimorbidity Index^1^

| **Condition** | **Weight** | **International Classification of Diseases, version 10**  **(ICD-10)** | **Anatomical Therapeutic Chemical Classification System (ATC)** |
| --- | --- | --- | --- |
| Secondary malignant neoplasms and malignancy of unspecified site | 22 | C76-C79, C80 |  |
| Malignant neoplasm of bronchus and lung | 19 | C34 |  |
| Alcoholic liver disease, liver fibrosis, cirrhosis, and failure | 13 | K70, K72, K74, K766-K767 |  |
| Mental and behavioral disorders due to use of alcohol | 12 | F10 |  |
| Decubitus ulcer and pressure area | 11 | L89 |  |
| Anti-dementia drugs | 11 |  | N06D |
| Chronic viral hepatitis | 10 | B18 |  |
| Dementia | 9 | F00-F03, G30 |  |
| Leukemia | 8 | C91-C95 |  |
| Malignant neoplasm of bladder | 8 | C67 |  |
| Drugs for constipation | 8 |  | A06A |
| Tumor of brain or meninge | 8 | C70, C71, C751-C753, D32, D330-D332, D352-D354, D42, D430-D432, D443-D445 |  |
| Multiple sclerosis | 7 | G35 |  |
| Other interstitial pulmonary diseases | 7 | J84 |  |
| Drugs used in opioid dependence | 7 |  | N07BC |
| Parkinson’s disease and other parkinsonism | 7 | G20-G22 |  |
| Antipsychotics | 7 |  | N05A excl. N05AN |
| Chronic kidney disease and unspecified kidney failure | 7 | N18-N19 |  |
| Volume depletion | 6 | E86 |  |
| Atherosclerosis, thrombosis, embolism, and other peripheral arterial disease | 5 | I70, I73-I74, I77 |  |
| Iron preparations | 5 |  | B03A |
| Antipropulsives | 5 |  | A07DA |
| Diseases of teeth and supporting structures | 5 |  | K02-K06, K08 |
| High-ceiling diuretics | 5 |  | C03C, C03EB |
| Long-acting anti-muscarinic agents | 5 |  | R03BB04-07 |
| Anaemia | 5 | D50-D59, D60-D64 |  |
| Malignant neoplasm of prostate | 5 | C61 |  |
| Epilepsy | 5 | G40-G41 |  |
| Insulins and analogues | 4 |  | A10A |
| Pneumonia | 4 | J12-J18 |  |
| Chronic lower respiratory diseases and failure | 4 | J41-J44, J47, J961, J969 |  |
| Digitalis glycosides | 4 |  | C01AA |
| Malignant neoplasm of breast | 4 | C50 |  |
| Cerebrovascular disease | 4 | I60-I69 |  |
| Aneurysm and dissection of aorta and other arteries | 4 | I71-I72 |  |
| Mental and behavioural disorders due to use of tobacco | 4 | F17 |  |
| Heart failure | 4 | I110, I130, I132, I420, I426-I429, I50 |  |
| Short-acting beta agonists | 3 |  | R03AC02-05 |
| Aldosterone antagonists | 3 |  | C03DA |
| Antidepressants | 3 |  | N06A |
| Opioids | 2 |  | N02A |
| Anilides | 2 |  | N02BE |
| Type 2 diabetes mellitus | 2 | E11 |  |
| Aortic and mitral valve disease | 2 | I05-I06, I34-I35 |  |
| Glucocorticoids for systemic use | 2 |  | H02AB |
| Platelet aggregation inhibitors excl. heparin | 2 |  | B01AC |
| Benzodiazepines and related drugs | 1 |  | N05BA, N05CD, N05CF |
| Beta-lactam antibacterials, penicillins | 1 |  | J01C |
| Angiotensin receptor blockers incl. combinations | -2 |  | C09C, C09D |
| Statins | -3 |  | C10AA |

## Table A.3. Single measures included in the social vulnerability index and their weights and time of measurement

| **Measure** | **Categories** | **Description** | **Data source and variable** | **Weight** | **Time of measurement** |
| --- | --- | --- | --- | --- | --- |
| Marital status | Unmarried | Never married, divorced, or widowed | The Population Registry, | 2 | Date of diagnosis |
|  | Married | Married or in a registered partnership |  | 0 |  |
| Ethnicity | Non-Danish | Immigrants and descendants | The Population Registry | 1 | Not applicable |
|  | Danish | Born in Denmark with Danish parents |  | 0 |  |
| Educational level | Short | ISCED 0–2: mandatory primary and lower secondary school | The Population’s Education Registry, variable hfaudd  Format: | 1 | Date of diagnosis |
|  | Medium | ISCED 3–4: optional upper secondary school and vocational education |  | 0.5 |  |
|  | Long | ISCED 5–8: bachelor degree, business academy education, or higher educational level |  | 0 |  |
| Equated disposable income | Low | < 1st quartile | Danish Income Statistics Registry (multiple variables), the Population Registry (variable family ID), and (Family Income Statistics, multiple variables)  Statistikbanken for the quartile of this variable in the general population by age group and calendar year | 2 | Calendar year prior to year of diagnosis |
|  | Medium | 1st to 3rd quartile |  | 0.5 |  |
|  | High | > 3rd quartile |  | 0 |  |
| Unemployment^a^ | Workforce detachment | Received unemployment benefits | DREAM, codes outlined in Table 3. | 2 | Exact year up to diagnosis |
|  | Employed | No unemployment benefits |  | 0 |  |
| Psychiatric comorbidity | Severe | One or more severe psychiatric comorbidity | Danish National Patient Registry (LPR-PSYK); ICD-10 codes outlined in Table 4. | 5 | Exact year up to diagnosis |
|  | Moderate | One or more moderate but no severe psychiatric comorbidity |  | 2 |  |
|  | None | No psychiatric diagnoses |  | 0 |  |
| Somatic comorbidity | Two or more | Two or more ICD-10 codes from the Elixhauser index | Danish National Patient Registry; ICD-10 codes outlined in Table 5 | 1 | Exact year up to diagnosis |
|  | One | One ICD-10 code from the Elixhauser index |  | 0.5 |  |
|  | None | No ICD-10 codes from the Elixhauser index |  | 0 |  |

^a^=As phrased by Møller et al.,^2^ but covers both temporary and permanent workforce detachment, including social assistance, unemployment compensations, immigrations benefit etc. (see Table A.4).

## Table A.4. DREAM codes used for rSVI unemployment and the study outcomes assessments.

| **Category** | **DREAM codes** | **Outcomes** |
| --- | --- | --- |
| Temporary unemployment benefits | 111-115, 121, 124-126, 211-219, 221, 231, 232, 299, 511, 522, 541, 759 | Out of workforce |
| Social assistance | 130-139, 151-153, 710-719, 730-739 | Out of workforce |
| Education or upskilling, not health related | 140-149, 222, 224, 225, 414, 720-729 | Out of workforce |
| Immigration benefits | 160, 163-169, 700, 703-709 | Out of workforce |
| Unemployed awaiting flexible job | 740-748 | Out of workforce |
| Rehabilitation | 750-758, 760, 763-768, 791, 792 | Out of workforce |
| Flex job | 622, 771-774, 779, 796 | Out of workforce |
| Disability pension | 783, 784, 793, 797, 781 | Disability pension |
| Disability pensioner in light-duty job | 761, 762, 769, 782 | Disability pension |
| Vocational rehabilitation program | 810, 813-818 | Out of workforce |
| Workability clarification | 785, 870, 873-878 | Out of workforce |
| Sick leave benefit^a^ | 890-899 | Sick leave |
| Parental leave ^b^ |  | N/A |

^a^ Includes long-term sick leave, defined as sick leaves lasting longer than 14-30 days, depending on the year.^3^ The variation is due to changes in the length of employer paid sick leave in Denmark (short term sick leave), before the government compensate the employer (long term sick leave).

**^b^** The original SVI considers women on parental leave as unemployed. We chose not to count parental leave, as this would categorize some women as socially vulnerable based on this criterion.

## Table A.5. ICD-10 codes used to classify psychiatric comorbidity in the rSVI

| **Diagnoses** | **ICD-10 codes** |
| --- | --- |
| ***Severe psychiatric comorbidity*** |  |
| Schizophrenia | F20.0-20.9 |
| Schizotypal disorder | F21.0-21.9 |
| Other psychoses | F22.0-29.9 |
| Manic and bipolar disorders | F30.0-31.9 |
| Borderline personality disorder | F60.3 |
| ***Moderate psychiatric comorbidity*** |  |
| Mild or moderate depression | F32.1, F32.2, F32.8-32.9, F33.1, F33.2, F33.4-33.9 |
| Severe depression | F32.2, F32.3, F33.2, F33.3 |
| Other affective disorders | F34.0-39.9 |
| Anxiety disorders | F40.0-41.9 |
| Obsessive-compulsive disorder | F42.0-42.9 |
| Reaction to severe stress and adjustment disorders | F43.0-43.9 |
| Eating disorders | F50.0-50.9 |
| Specific personality disorders | F60.0-60.30, F60.32-62.9 |
| Attention-deficit hyperactivity disorders | F90.0-90.9, F98.8C |
| Conduct disorders | F91.0-92.9 |

## Table A.6. ICD-10 codes used for the Elixhauser Comorbidity Index^4^

| **Comorbidity** | **ICD-10 Codes** |
| --- | --- |
| Congestive heart failure | I09.9, I11.0, I13.0, I13.2, I25.5, I42.0, I42.5 - I42.9, I43.x, I50.x, P29.0 |
| Cardiac arrhythmias | I44.1 - I44.3, I45.6, I45.9, I47.x - I49.x, R00.0, R00.1, R00.8, T82.1, Z45.0, Z95.0 |
| Valvular disease | A52.0, I05.x - I08.x, I09.1, I09.8, I34.x - I39.x, Q23.0 - Q23.3, Z95.2 - Z95.4 |
| Pulmonary circulation disorders | I26.x, I27.x, I28.0, I28.8, I28.9 |
| Peripheral vascular disorders | I70.x, I71.x, I73.1, I73.8, I73.9, I77.1, I79.0, I79.2, K55.1, K55.8, K55.9, Z95.8, Z95.9 |
| Hypertension, uncomplicated | I10.x |
| Hypertension, complicated | I11.x - I13.x, I15.x |
| Paralysis | G04.1, G11.4, G80.1, G80.2, G81.x, G82.x, G83.0 - G83.4, G83.9 |
| Other neurological disorders | G10.x - G13.x, G20.x - G22.x, G25.4, G25.5, G31.2, G31.8, G31.9, G32.x,  G35.x - G37.x, G40.x, G41.x, G93.1, G93.4, R47.0, R56.x |
| Chronic pulmonary disease | I27.8, I27.9, J40.x - J47.x, J60.x - J67.x, J68.4, J70.1, J70.3 |
| Diabetes, uncomplicated | E10.0, E10.1, E10.9, E11.0, E11.1, E11.9, E12.0, E12.1, E12.9, E13.0, E13.1, E13.9, E14.0, E14.1, E14.9 |
| Diabetes, complicated | E10.2 - E10.8, E11.2 - E11.8, E12.2 - E12.8, E13.2 - E13.8, E14.2 - E14.8 |
| Hypothyroidism | E00.x - E03.x, E89.0 |
| Liver disease | B18.x, I85.x, I86.4, I98.2, K70.x, K71.1, K71.3 - K71.5, K71.7, K72.x - K74.x,  K76.0, K76.2 - K76.9, Z94.4 |
| Peptic ulcer disease, excluding bleeding | K25.7, K25.9, K26.7, K26.9, K27.7, K27.9, K28.7, K28.9 |
| AIDS/HIV | B20.x - B22.x, B24.x |
| Lymphoma | C81.x - C85.x, C88.x, C96.x, C90.0, C90.2 |
| Metastatic cancer | C77.x - C80.x |
| Solid tumour without metastasis | C00.x - C26.x, C30.x - C34.x, C37.x - C41.x, C43.x, C45.x - C58.x, C60.x - C76.x, C97.x |
| Rheumatoid arthritis/collagen vascular diseases | L94.0, L94.1, L94.3, M05.x, M06.x, M08.x, M12.0, M12.3, M30.x, M31.0 - M31.3, M32.x - M35.x, M45.x, M46.1, M46.8, M46.9 |
| Coagulopathy | D65 - D68.x, D69.1, D69.3 - D69.6 |
| Obesity | E66.x |
| Weight loss | E40.x - E46.x, R63.4, R64 |
| Fluid and electrolyte disorders | E22.2, E86.x, E87.x |
| Blood loss anaemia | D50.0 |
| Deficiency anaemia | D50.8, D50.9, D51.x - D53.x |
| Alcohol abuse | F10, E52, G62.1, I42.6, K29.2, K70.0, K70.3, K70.9, T51.x, Z50.2, Z71.4, Z72.1 |
| Drug abuse | F11.x - F16.x, F18.x, F19.x, Z71.5, Z72.2 |
| Psychoses | F20.x, F22.x - F25.x, F28.x, F29.x, F30.2, F31.2, F31.5 |
| Depression | F20.4, F31.3 - F31.5, F32.x, F33.x, F34.1, F41.2, F43.2 |

## Figure A.2. Sick leave among breast cancer patients compared to matched comparisons, restricted to breast cancer patients assigned intention-to-treat A) chemotherapy and B) endocrine therapy

A:


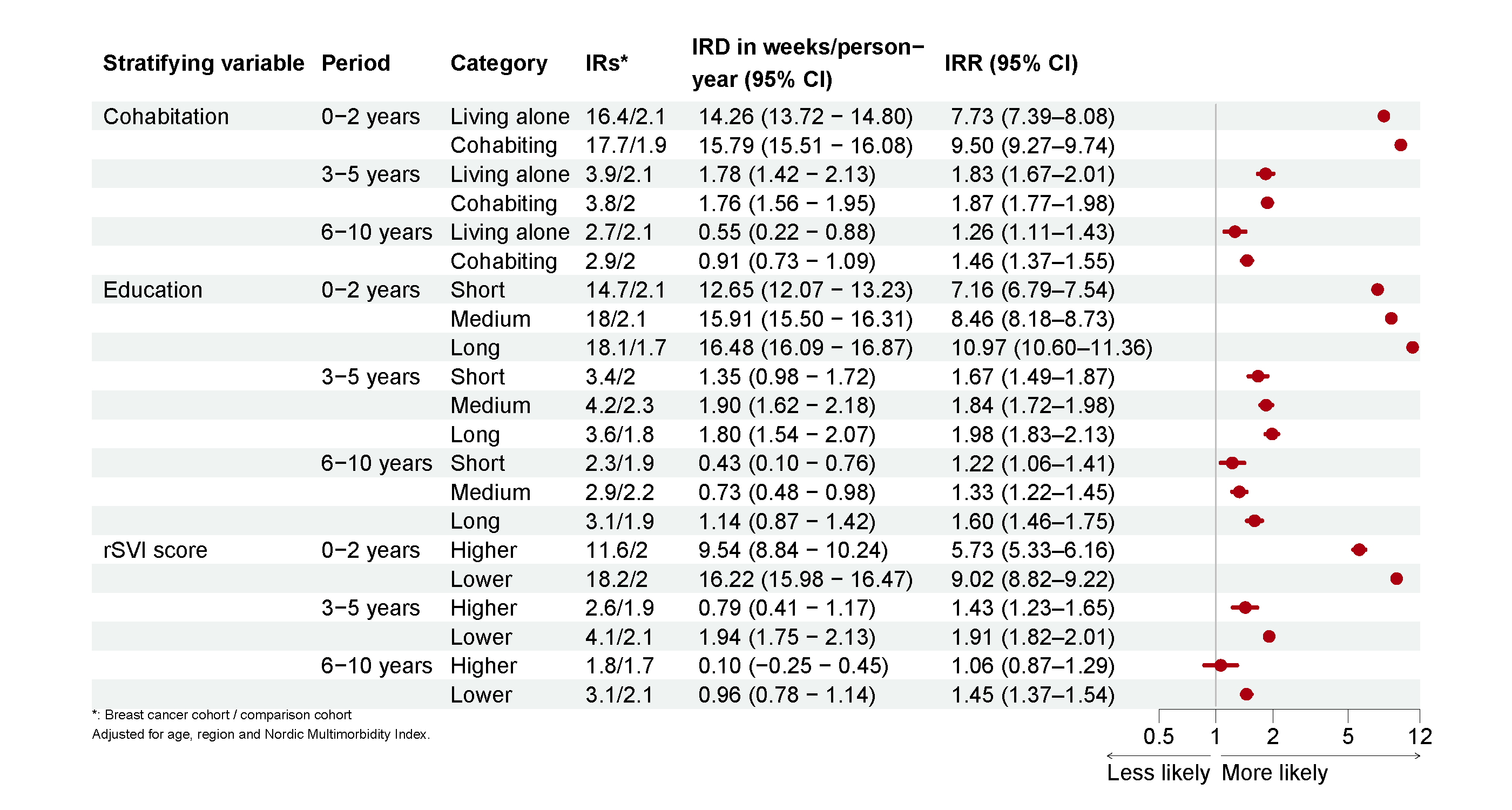


B:
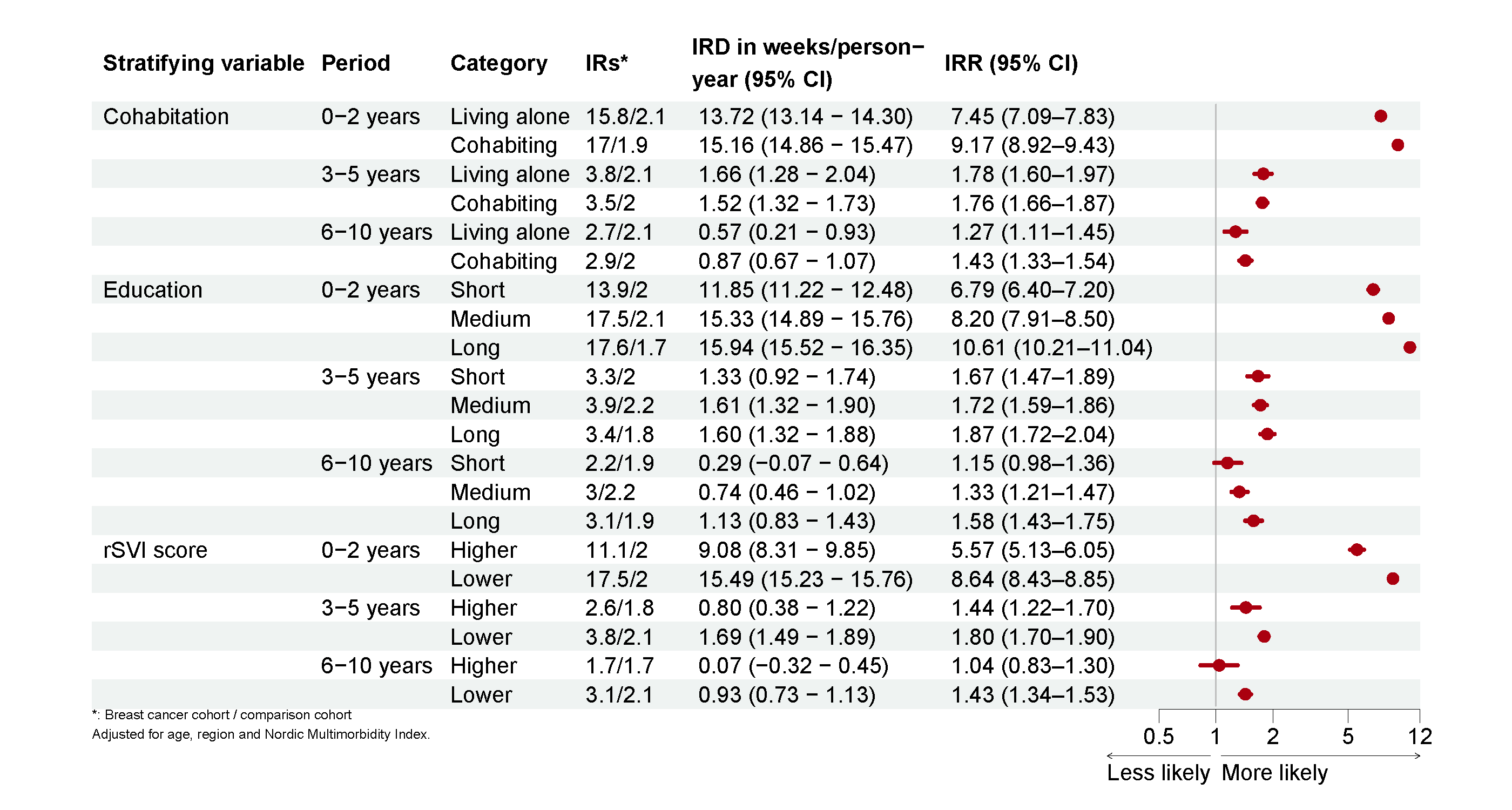


## Figure A.3. Workforce detachment among breast cancer patients compared to matched comparisons, restricted to breast cancer patients assigned intention-to-treat A) chemotherapy and B) endocrine therapy

A:

##
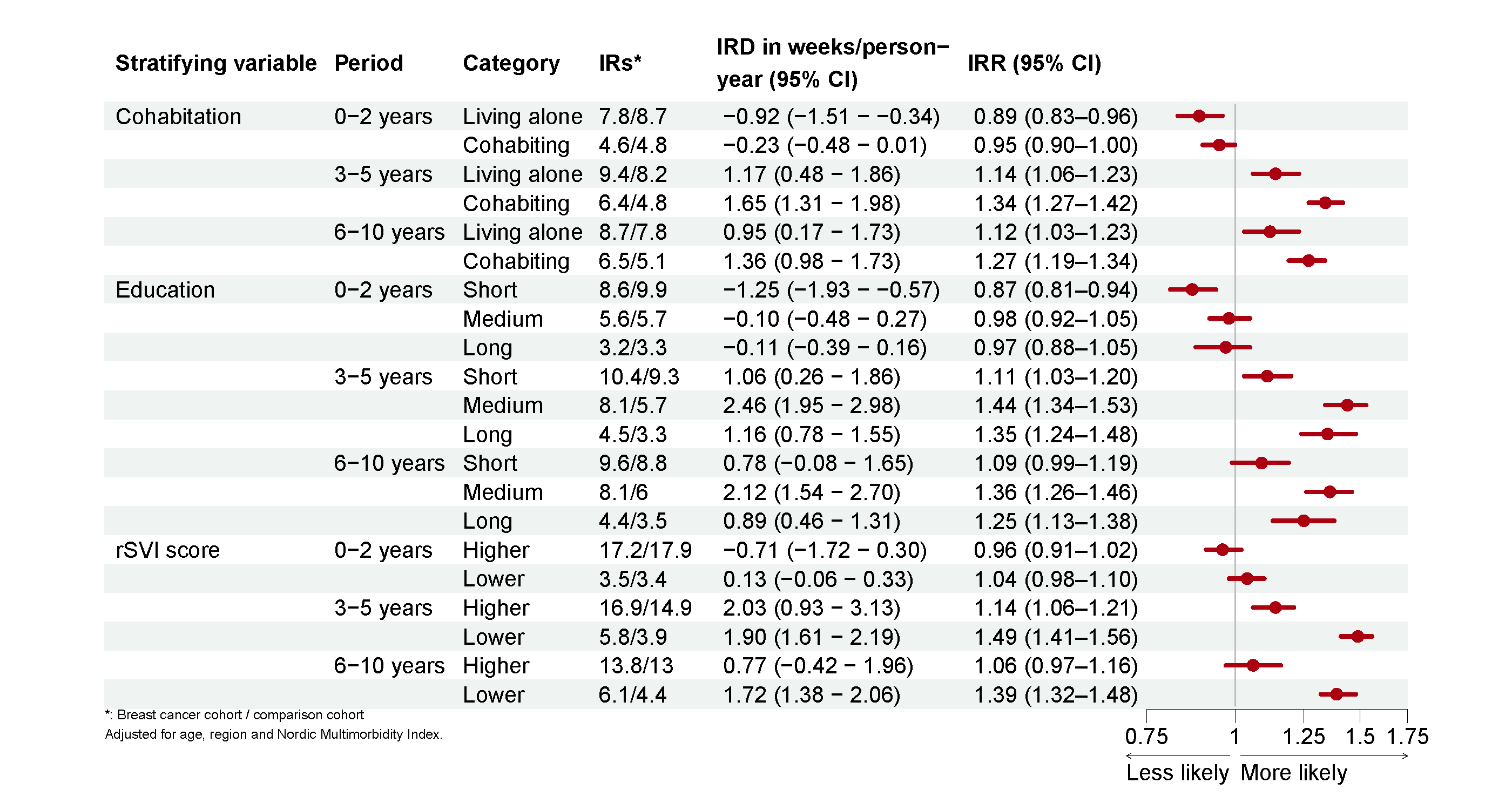


B:
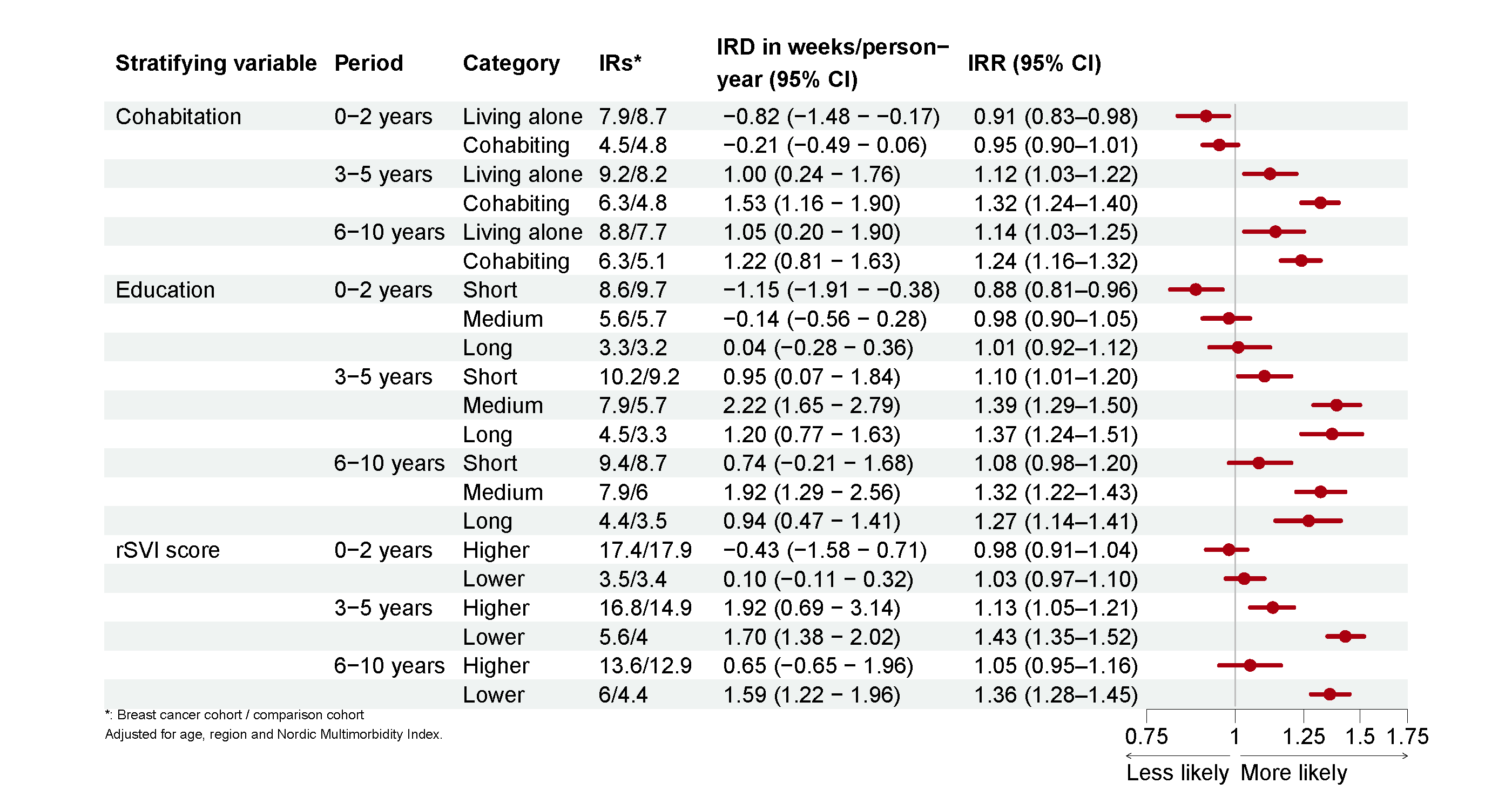


## Figure A.4. Disability pension among breast cancer patients compared to matched comparisons, restricted to breast cancer patients assigned intention-to-treat A) chemotherapy and B) endocrine therapy

A:


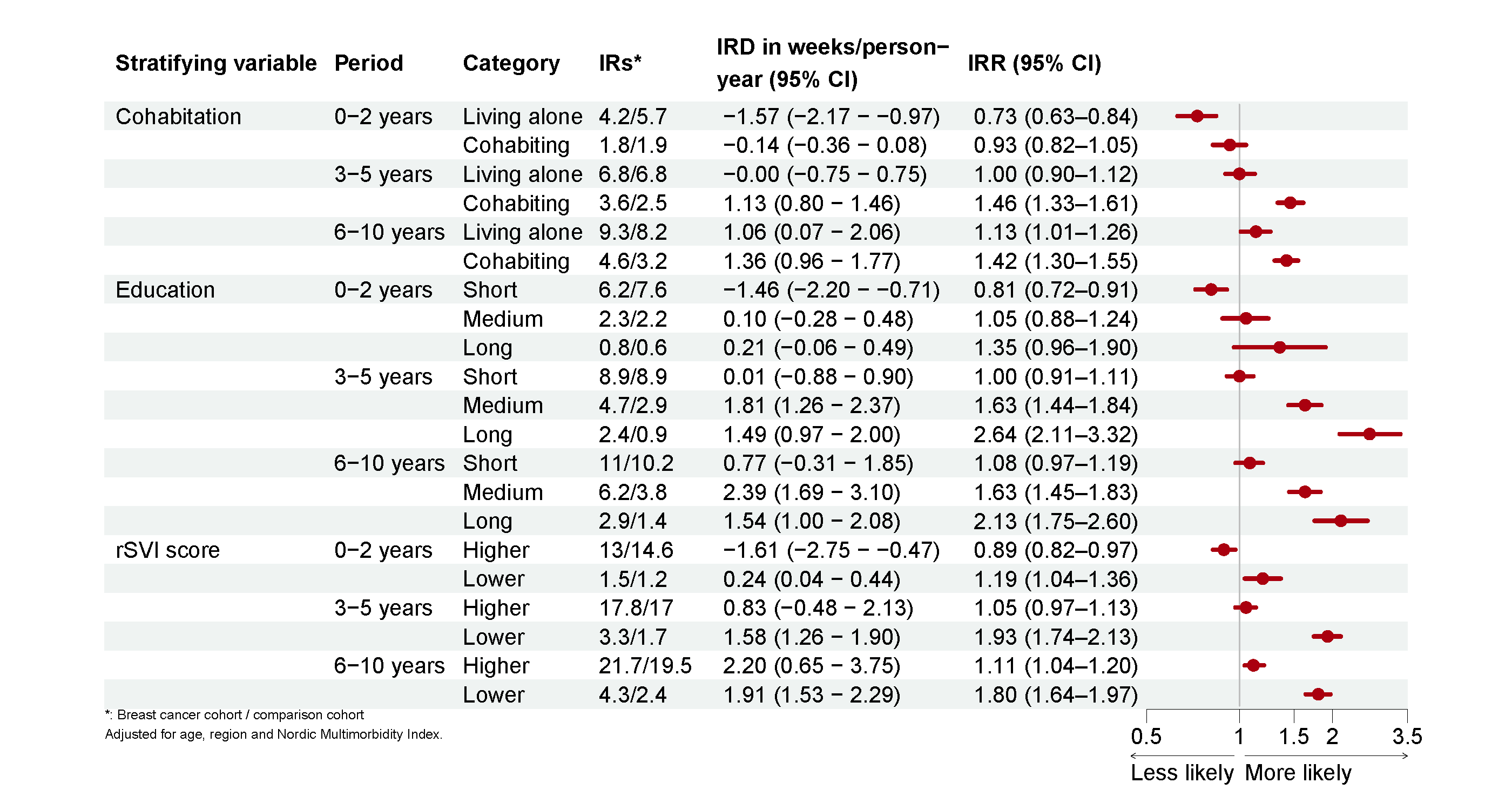


B:

##
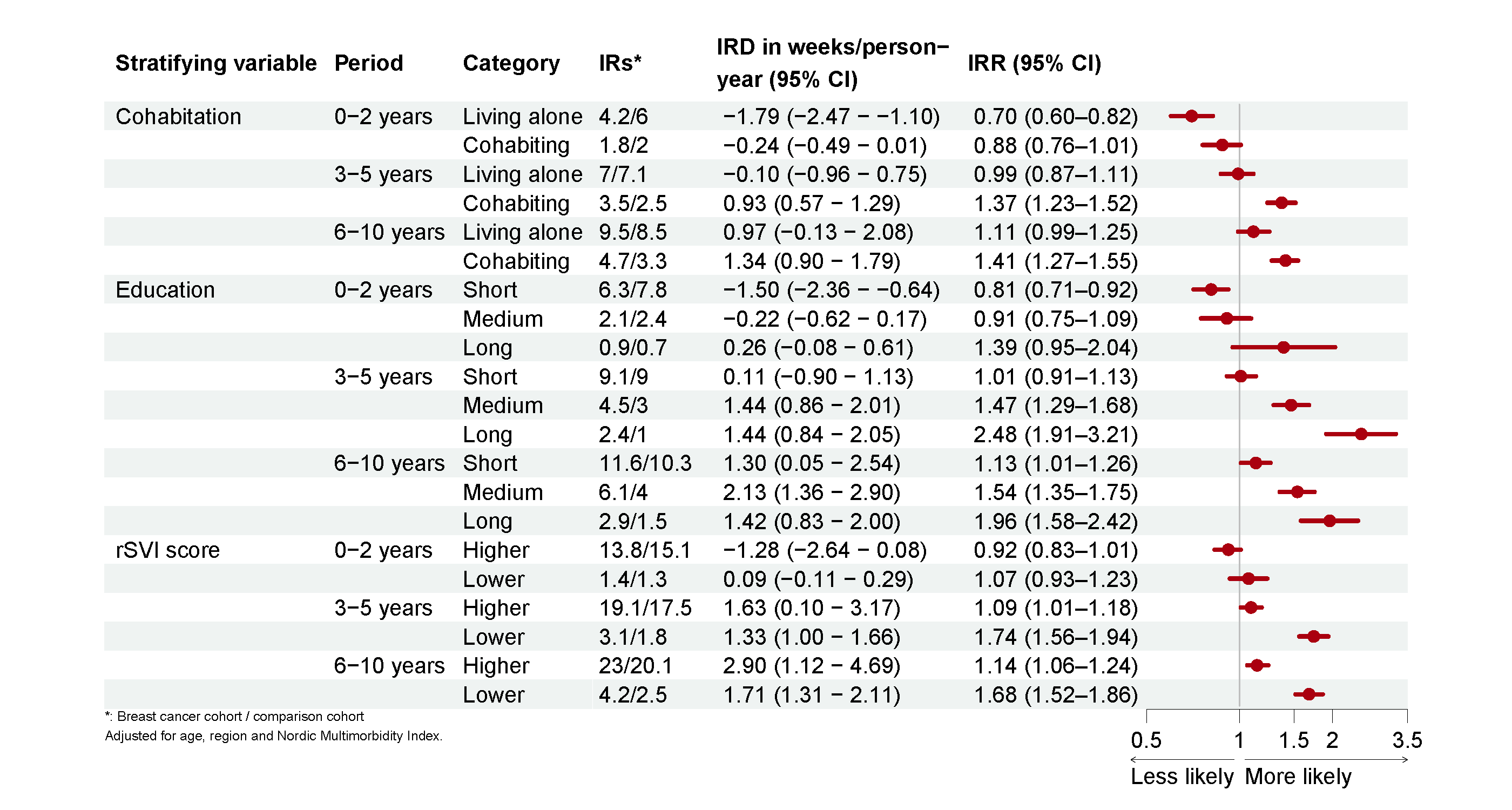


## Figure A.5. Sick leave, workforce detachment and disability pension among breast cancer patients compared to matched comparisons, restricted to women employed prior to index

##
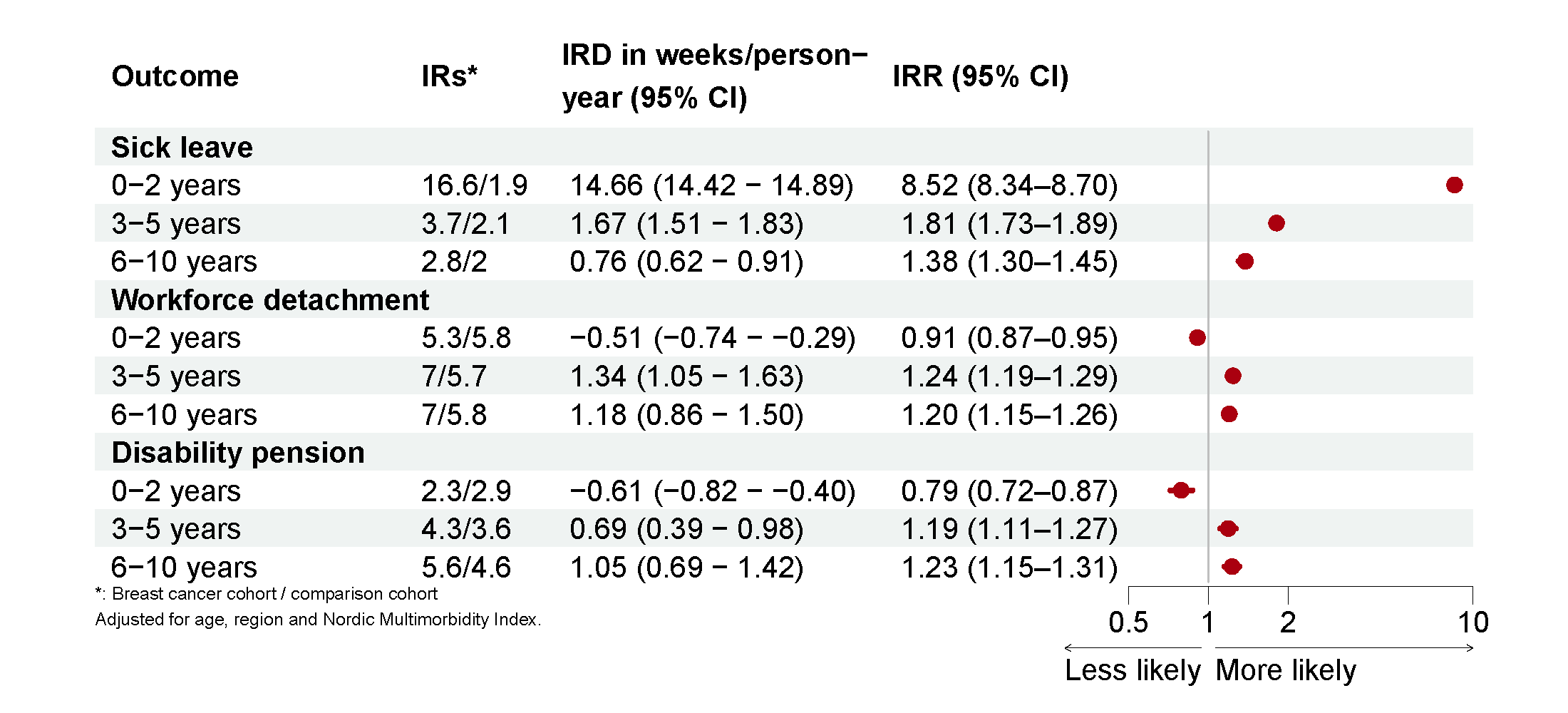


## Figure A.6. Sick leave among employed breast cancer patients compared to matched comparisons, stratified by cohabitation, education and rSVI.

##
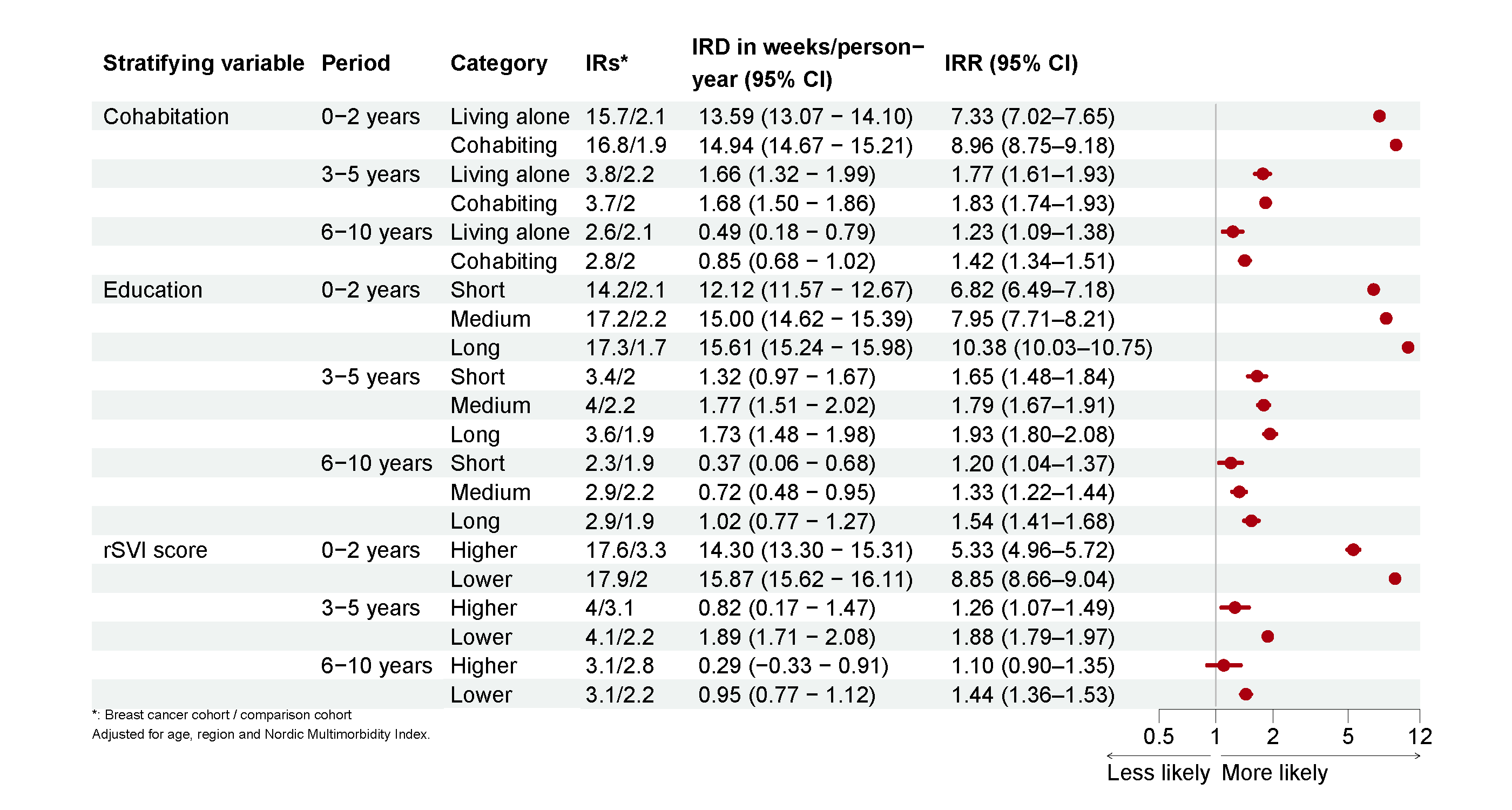


## Figure A.7. Workforce detachment among employed breast cancer patients compared to matched comparisons, stratified by cohabitation, education and rSVI.

##
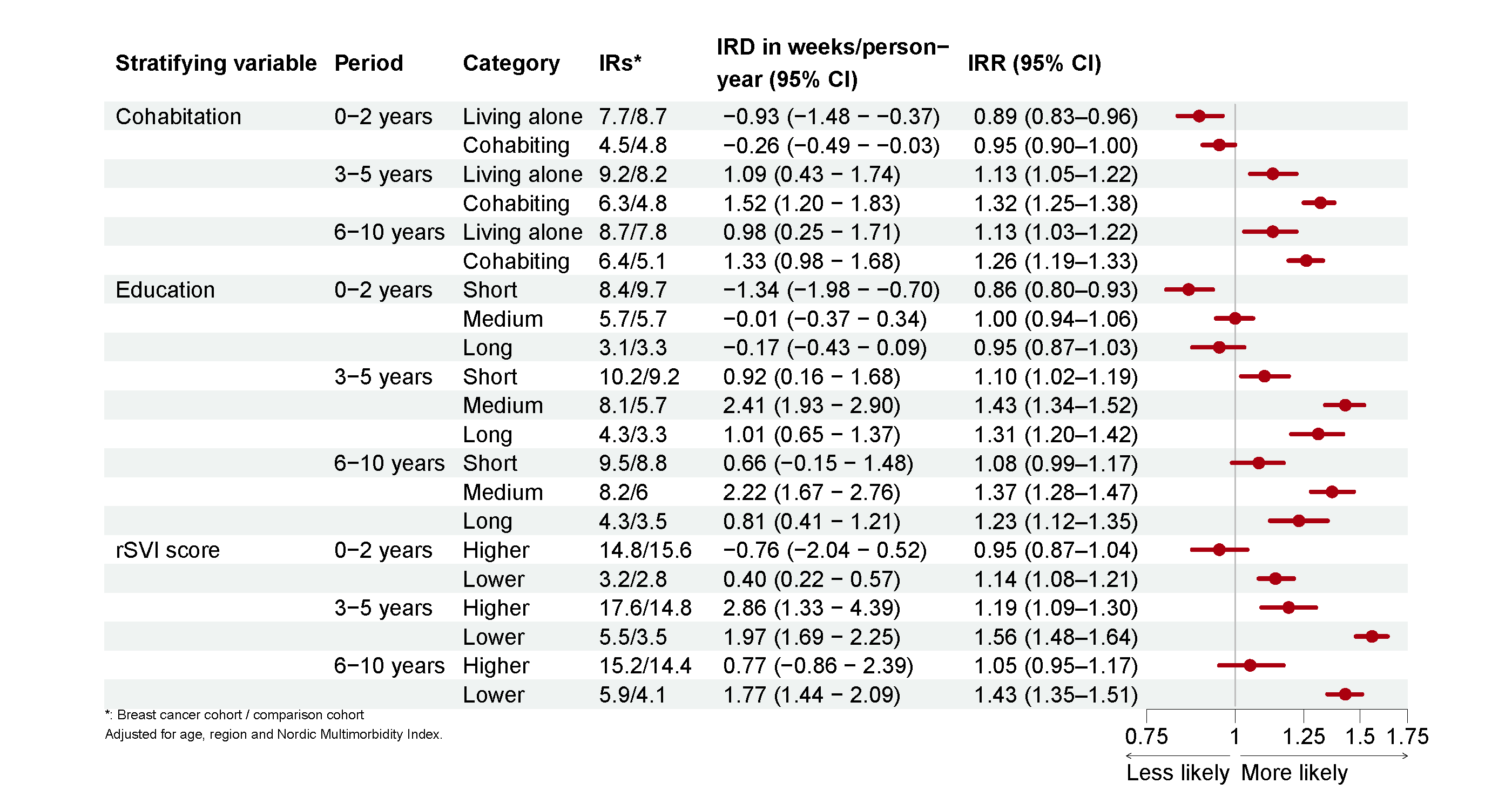


## Figure A.8. Disability pension among employed breast cancer patients compared to matched comparisons, stratified by cohabitation, education and rSVI.


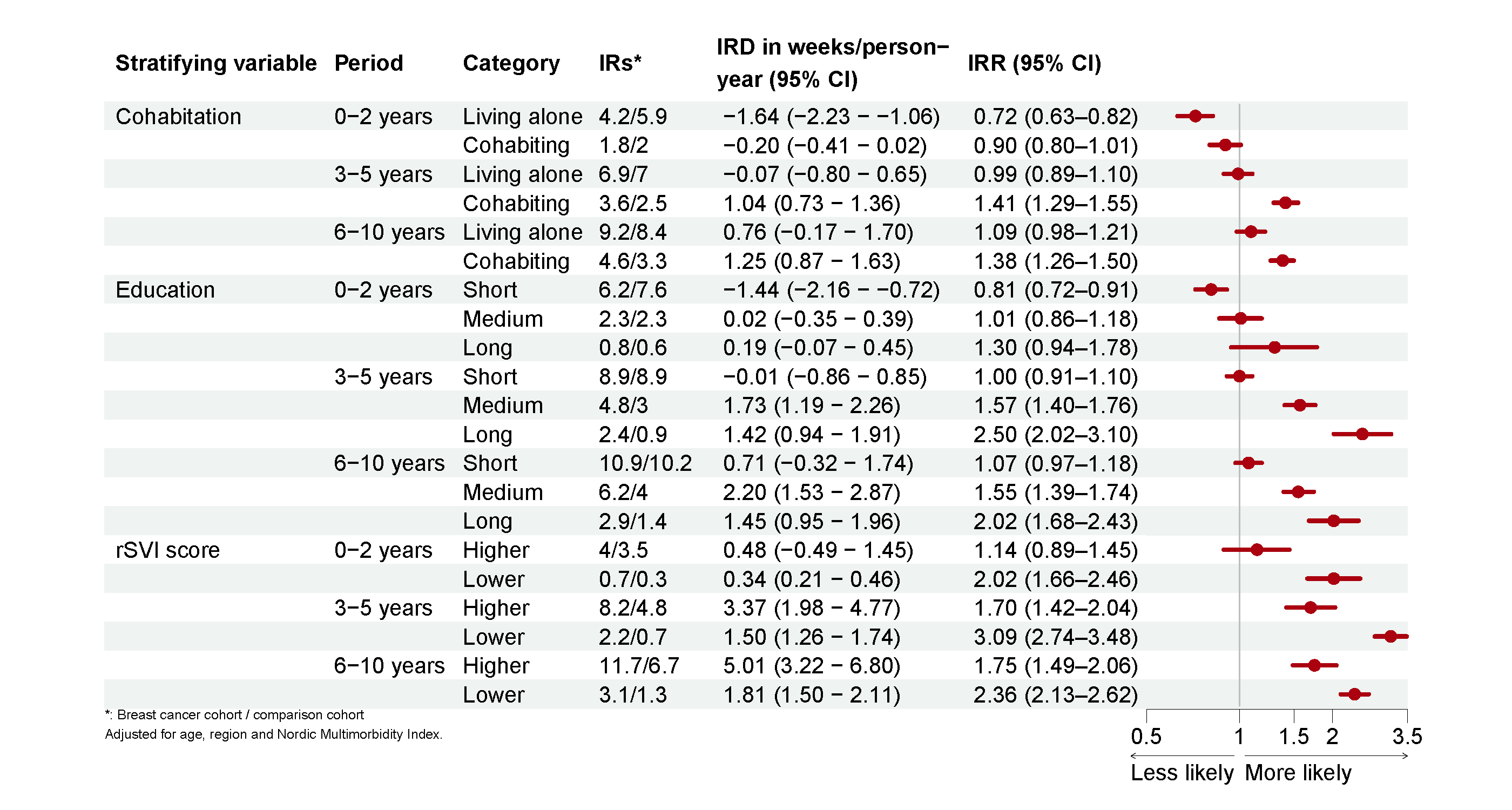


## Figure A.9. Annual incidence rate per. 1,000 person years of disability pension in women with breast cancer and the general population


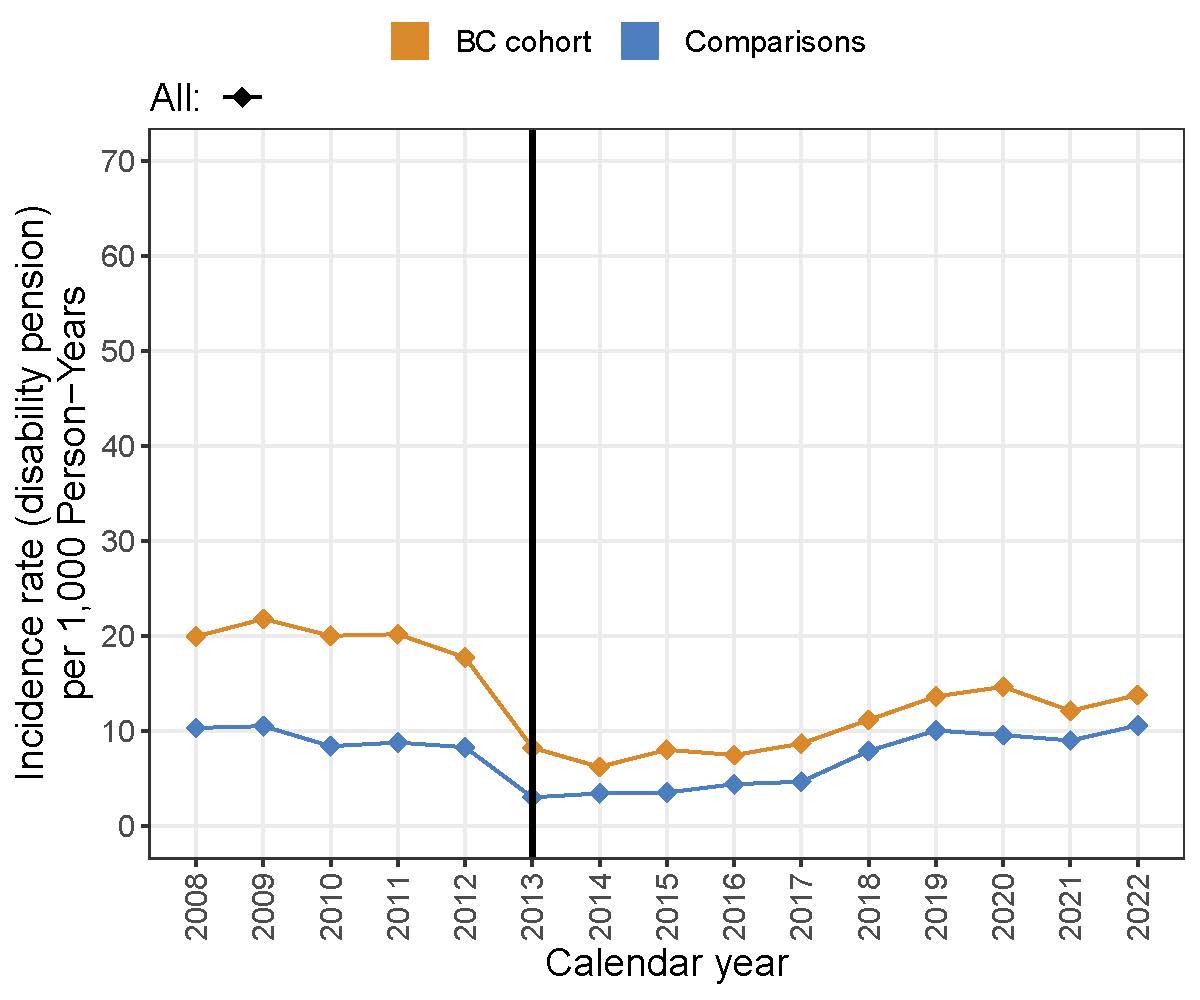


## Figure A.10. Annual incidence rate per. 1,000 person years of disability pension in women with breast cancer and the general population by age, cohabitation status, education and social vulnerability


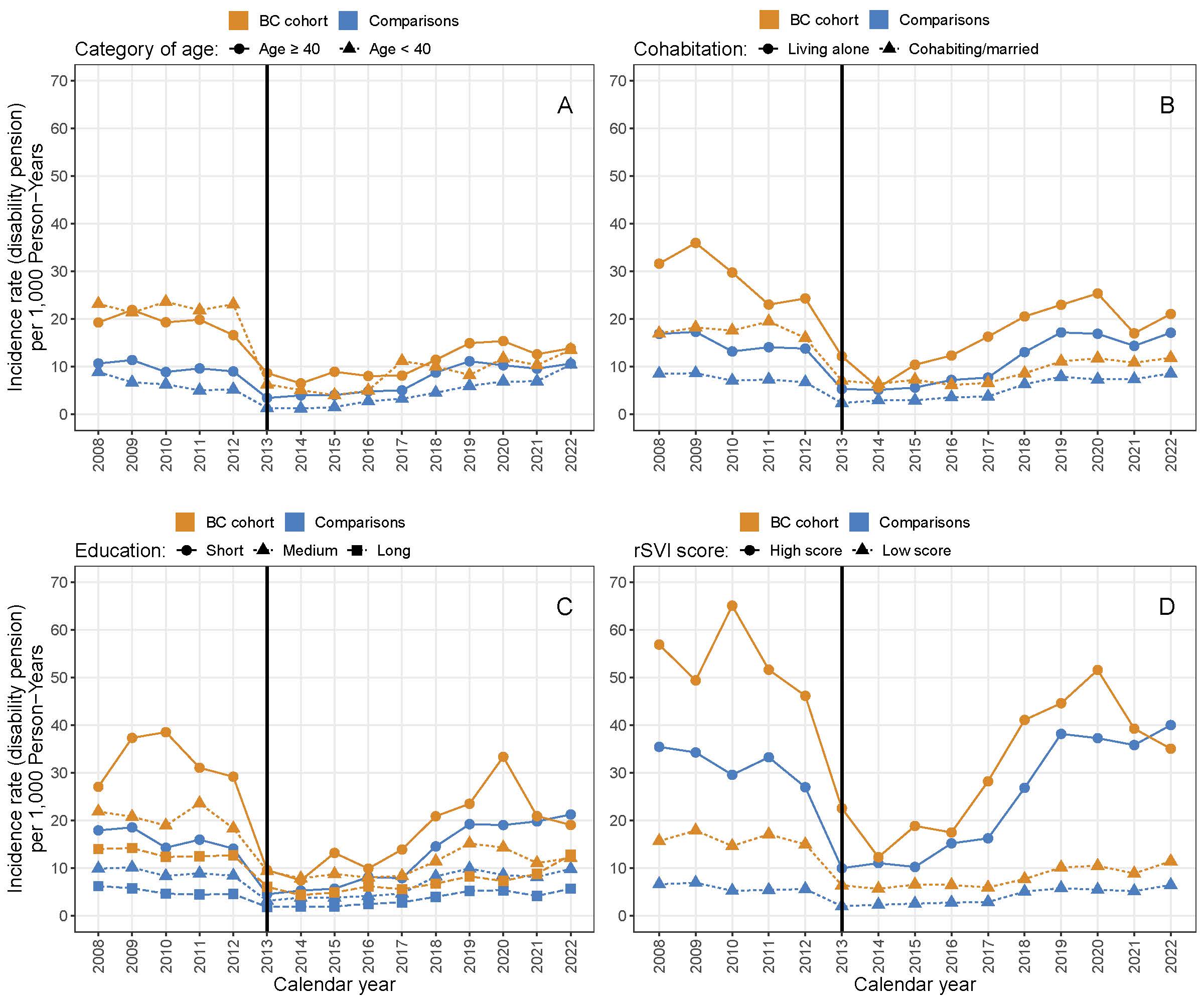


**References**

1. Kristensen KB, Lund LC, Jensen PB, et al. Development and Validation of a Nordic Multimorbidity Index Based on Hospital Diagnoses and Filled Prescriptions. *Clin Epidemiol*. 2022;14:567-579. doi:10.2147/CLEP.S353398

2. Møller JJK, la Cour K, Pilegaard MS, Möller S, Jarlbaek L. Identification of socially vulnerable cancer patients - development of a register-based index (rSVI). *Support Care Cancer Off J Multinatl Assoc Support Care Cancer*. 2022;30(6):5277-5287. doi:10.1007/s00520-022-06937-3

3. Hjorth CF, Kjærulff TM, Thomsen MK, et al. SEPLINE: Socioeconomic Position in Epidemiological Research-A National Guideline on Danish Registry Data. *Clin Epidemiol*. 2025;17:593-624. doi:10.2147/CLEP.S520772

4. Elixhauser A, Steiner C, Harris DR, Coffey RM. Comorbidity measures for use with administrative data. *Med Care*. 1998;36(1):8-27. doi:10.1097/00005650-199801000-00004
